# Supplementary material for: Extracellular Fluid Flow Induces Shallow Quiescence Through Physical and Biochemical Cues
Source: Front Cell Dev Biol. 2022 Feb 24;10:792719. doi: 10.3389/fcell.2022.792719 (PMC8912726; doi:10.3389/fcell.2022.792719)
Supplement: Supplementary file 1 [file DataSheet1.pdf]

Supplementary Material for

Extracellular Fluid Flow Induces Shallow Quiescence through Physical and Biochemical Cues

Bi Liu[1,2], Xia Wang[2,3], Linan Jiang[4], Jianhua Xu[1], Yitshak Zohar[4] and Guang Yao[2]

[1]School of Pharmacy, Fujian Provincial Key Laboratory of Natural Medicine Pharmacology, Fujian Medical University, Fuzhou, China
[2]Department of Molecular and Cellular Biology, University of Arizona, Tucson, AZ, United States
[3]College of Animal Science and Technology, Northwest A&F University, Yangling, China
[4]Aerospace and Mechanical Engineering, University of Arizona, Tucson, AZ, United States

This PDF file includes:

Figures S1 to S3
Tables S1 to S2

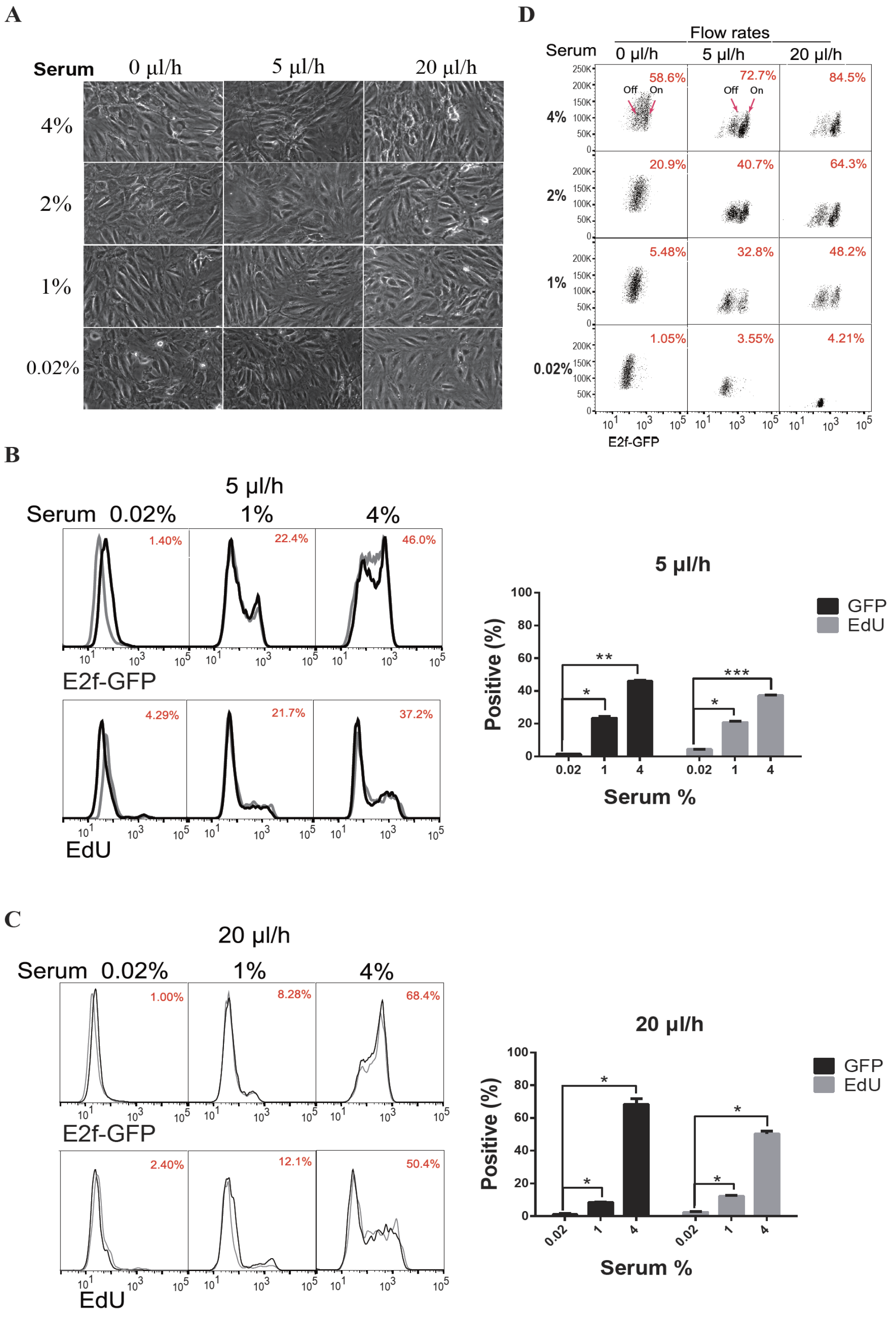

Fig S1. Experimental system configuration and validation. (**A**) Cell morphology under varying medium flow rates. REF/E23 cells seeded in microfluidic devices were induced to and maintained in quiescence by culturing them in serum-starvation medium for 4 days under the indicated flow rates, then either remained in quiescence (0.02% serum) or stimulated with serum at the indicated concentrations (1-4%) for 26 hours. Phase-contrast images were taken with a 20x objective lens. (**B, C**) E2f-GFP and EdU-incorporation readouts of cellular quiescence and cell cycle reentry. REF/E23 cells were induced to quiescence as in (A) under a medium flow rate of 5 ul/h (B) or 20 ul/h (C), and then stimulated with serum at the indicated concentrations. Cells were harvested after 26 and 30 hours of simulation, respectively, for E2f-GFP and EdU assays. (Left) Numbers in red indicate the average E2f-On% or EdU+% as indicated from duplicate samples (black and grey histograms). (Right) Statistic bar chart of E2f-On% and EdU+% from the left-panel histograms. Error bars, SEM (n = 2), * $p < 0.05$, ** $p < 0.01$, *** $p < 0.001$. (**D**) Dot plots of Fig 3A. Y-axis, forward-scatter; x-axis, E2f-GFP.

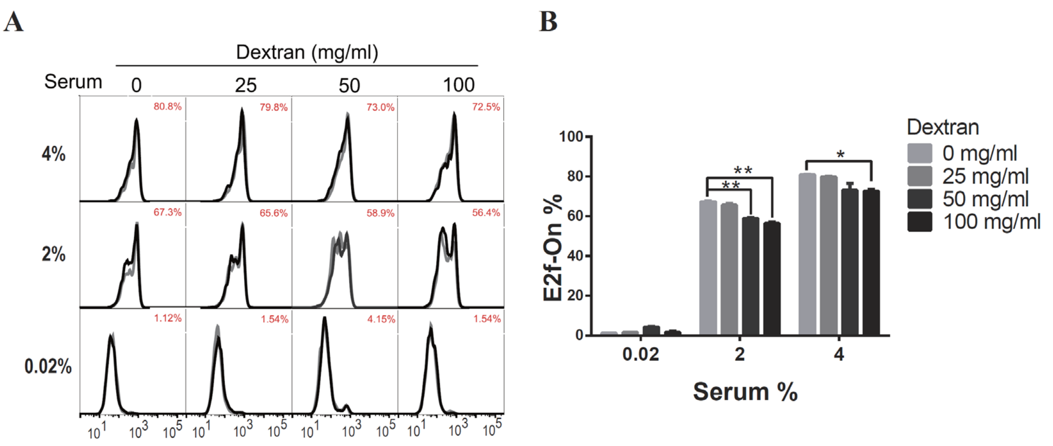

Fig S2. The effect of varying dextran concentrations on quiescence depth in static culture. REF/E23 cells were induced to and maintained in quiescence by culturing them in static serum-starvation medium (in well plate) for 4 days with dextran at the indicated concentrations. Cells were subsequently stimulated with serum at the indicated concentrations for 26 hours, and the E2f-On% were assayed. (**A**) E2f-GFP histograms with red numbers indicating the average E2f-On% from duplicate samples (black and grey). (**B**) Statistic bar chart of the E2f-On% in cell populations (from A) as a function of dextran concentration (in serum-starvation) and serum concentration (in serum-stimulation). Bar graphs showing the E2f-On% from the left panels. Error bars, SEM ($n = 2$), * $p < 0.05$, ** $p < 0.01$.

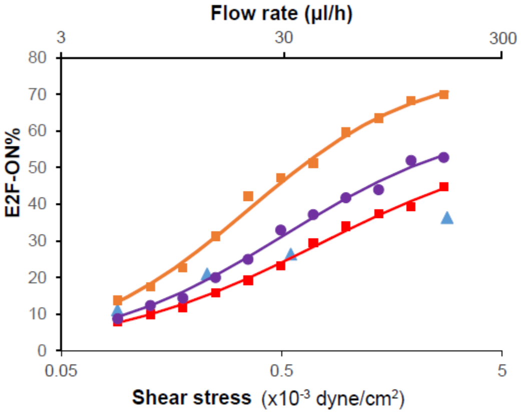

Fig S3. Simulation results on the effects of shear stress and extracellular factor replacement on quiescence depth. Simulations of fluid flow rate effects (orange) are the same as in Fig. 6B. Simulations of the effects of shear stress (red) and extracellular factor replacement (purple) were performed in the same way as the simulations of fluid flow rate effects (orange), except that the FR term in Table S1 was multiplied by 0.75 and 0.85, respectively. Blue triangle, the average E2f-On% in response to 2% serum stimulation, in cells under 5 ul/h extracellular fluid flow with the indicated shear stress level during serum starvation (as in Fig. 6B, based on Fig. 4A and Table 1). Each solid curve represents the best fit of simulation data points to a Hill function.

Table S1. The Rb-E2f bistable switch model with fluid-flow effects (adapted from (Yao et al., 2008), with modifications marked with •)

$\frac{d[M]}{dt} = k_M \left(\frac{[S]}{K_S + [S]} + FR \right) ss \bullet - d_M[M]$
$\frac{d[CD]}{dt} = \frac{k_{CD}[M]}{K_M + [M]} + k_{CDS} \left(\frac{[S]}{K_S + [S]} + FR \right) ss \bullet - d_{CD}[CD]$
$\frac{d[R]}{dt} = k_R + \frac{k_{DP}[RP]}{K_{RP} + [RP]} - k_{RE}[R][E] - \frac{k_{P1}[CD][R]}{K_{CD} + [R]} - \frac{k_{P2}[CE][R]}{K_{CE} + [R]} - d_R[R]$
$\frac{d[CE]}{dt} = \frac{k_{CE}[E]}{K_E + [E]} - d_{CE}[CE]$
$\frac{d[E]}{dt} = k_E \left(\frac{[M]}{K_M + [M]} \right) \left(\frac{[E]}{K_E + [E]} \right) + \frac{k_b[M]}{K_M + [M]} + \frac{k_{P1}[CD][RE]}{K_{CD} + [RE]} + \frac{k_{P2}[CE][RE]}{K_{CE} + [RE]} - k_{RE}[R][E] - d_E[E]$
$\frac{d[RP]}{dt} = \frac{k_{P1}[CD][R]}{K_{CD} + [R]} + \frac{k_{P2}[CE][R]}{K_{CE} + [R]} + \frac{k_{P1}[CD][RE]}{K_{CD} + [RE]} + \frac{k_{P2}[CE][RE]}{K_{CE} + [RE]} - \frac{k_{DP}[RP]}{K_{RP} + [RP]} - d_{RP}[RP]$
$\frac{d[RE]}{dt} = k_{RE}[R][E] - \frac{k_{P1}[CD][RE]}{K_{CD} + [RE]} - \frac{k_{P2}[CE][RE]}{K_{CE} + [RE]} - d_{RE}[RE]$
$FR = w * \frac{f_0}{K_f + f_0} \bullet$

<u>Variables:</u>

S: serum concentration; *M*: Myc; *E*: E2F; *CD*: Cyclin D/Cdk4,6; *CE*: Cyclin E/Cdk2; *R*: Rb family proteins; *RP*: Phosphorylated Rb; *RE*: Rb-E2F complex; *FR*, extracellular fluid flow effects •; f_0: extracellular fluid flow rate •

<u>Initial condition:</u>

[*M*] = [*E*] = [*CD*] = [*CE*] =[*R*] = [*RP*] = 0 nM; [*RE*] = 0.55 nM;
f_0 = 0, 5, 20 μl hr^{-1} •

Note: Model parameters are adapted from (Yao *et al.*, 2008) and defined in Table S2, including newly added parameters.

Table S2. Model parameters (adapted from (Yao *et al.*, 2008), with modifications marked with •)

Symbol	Values	Description
k_M	1.0 nM hr^{-1}	Rate constant of Myc synthesis driven by growth factors
k_E	0.4 nM hr^{-1}	Rate constant of E2F synthesis driven by Myc and E2F
k_b	0.003 nM hr^{-1}	Rate constant of E2F synthesis driven by Myc alone
k_{CD}	0.03 nM hr^{-1}	Rate constant of CycD synthesis driven by Myc
k_{CDS}	0.45 nM hr^{-1}	Rate constant of CycD synthesis driven by growth factors
k_{CE}	0.35 nM hr^{-1}	Rate constant of CycE synthesis driven by E2F
k_R	0.18 nM hr^{-1}	Rate constant of Rb constitutive synthesis
k_{P1}	18 hr^{-1}	Phosphorylation rate constant of Rb by CycD/Cdk4,6
k_{P2}	18 hr^{-1}	Phosphorylation rate constant of Rb by CycE/Cdk2
k_{DP}	3.6 nM hr^{-1}	Dephosphorylation rate constant of Rb by phosphatases
k_{RE}	180 nM^{-1} hr^{-1}	Association rate constant of Rb and E2F
K_S	2.5 nM	Michaelis-Menten parameter for CycD and Myc synthesis by growth factors
K_E	0.15 nM	Michaelis-Menten parameter for CycE and E2F synthesis by E2F
K_M	0.15 nM	Michaelis-Menten parameter for CycD and E2F synthesis by Myc
K_{RP}	0.01 nM	Michaelis-Menten parameter for Rb dephosphorylation
K_{CD}	0.92 nM	Michaelis-Menten parameter for Rb phosphorylation by CycD/Cdk4,6
K_{CE}	0.92 nM	Michaelis-Menten parameter for Rb phosphorylation by CycE/Cdk2
d_M	0.7 hr^{-1}	Degradation rate constant of Myc
d_E	0.25 hr^{-1}	Degradation rate constant of E2F
d_{CD}	1.5 hr^{-1}	Degradation rate constant of CycD
d_{CE}	1.5 hr^{-1}	Degradation rate constant of CycE
d_R	0.06 hr^{-1}	Degradation rate constant of Rb
d_{RP}	0.06 hr^{-1}	Degradation rate constant of phosphorylated Rb
d_{RE}	0.03 hr^{-1}	Degradation rate constant of Rb-E2F complex
K_f	2.5-15.0 μl hr^{-1}	*Michaelis-Menten parameter for the effects of fluid flow •
w	0.2-0.3	*Scaling factor for the effects of fluid flow •
ss	0.80	Scaling factor reflecting the batch variations of individual experiments •

*Serum concentration-dependent as follows:

[S] = 0.02 or 1: w = 0.3, *Kf* = 2.5

[S] = 2: w = 0.3, *Kf* = 13

[S] = 4: w = 0.2, *Kf* = 15

Supplementary References

Yao, G., Lee, T.J., Mori, S., Nevins, J.R., and You, L. (2008). A bistable Rb-E2F switch underlies the restriction point. Nat Cell Biol *10*, 476-482. 10.1038/ncb1711.
